# Symptoms and Subjective Quality of Life in Post-Traumatic Stress Disorder: A Longitudinal Study

**DOI:** 10.1371/journal.pone.0060991

**Published:** 2013-04-09

**Authors:** Domenico Giacco, Aleksandra Matanov, Stefan Priebe

**Affiliations:** 1 Unit for Social and Community Psychiatry, Barts and the London School of Medicine and Dentistry, Queen Mary University of London, London, United Kingdom; 2 Department of Psychiatry, University of Naples SUN, Naples, Italy; Max Planck Institute of Psychiatry, Germany

## Abstract

**Background:**

Evidence suggests that post-traumatic stress disorder (PTSD) is associated with substantially reduced subjective quality of life (SQOL). This study aimed to explore whether and how changes in the levels of PTSD symptom clusters of intrusion, avoidance and hyperarousal are associated with changes in SQOL.

**Methods:**

Two samples with PTSD following the war in former Yugoslavia were studied, i.e. a representative sample of 530 people in five Balkan countries and a non-representative sample of 215 refugees in three Western European countries. They were assessed on average eight years after the war and re-interviewed one year later. PTSD symptoms were assessed on the Impact of Event Scale - Revised and SQOL on the Manchester Short Assessment of Quality of Life. Linear regression and a two-wave cross lagged panel analysis were used to explore the association between PTSD symptom clusters and SQOL.

**Results:**

The findings in the two samples were consistent. Symptom reduction over time was associated with improved SQOL. In multivariable analyses adjusted for the influence of all three clusters, gender and time since war exposure, only changes in hyperarousal symptoms were significantly associated with changes in SQOL. The two-wave cross-lagged panel analysis suggested that the link between hyperarousal symptoms and SQOL is bidirectional.

**Conclusions:**

Low SQOL of patients with war-related PTSD is particularly associated with hyperarousal symptoms. The findings suggest a bidirectional influence: a reduction in hyperarousal symptoms may result in improved SQOL, and improvements in SQOL may lead to reduced hyperarousal symptoms.

## Introduction

Patients with post-traumatic stress disorder (PTSD) report a poorer subjective quality of life (SQOL) than patients with other anxiety disorders [1]–[3].

In recent years, SQOL has become a widely established patient-reported outcome in severe mental disorders [4]–[6]. The most established definition of SQOL is based on the Lehman’s approach [7], which considers SQOL as the patient’s satisfaction with life in general and with a number of major life domains.

The negative correlation between general PTSD symptom levels and subjective quality of life has been shown both in cross-sectional [1], [8] and longitudinal studies [9]–[10].

However, to our knowledge, only two studies [8], [11], carried out in relatively small and non-representative samples, have investigated which symptom clusters of PTSD (defined according to DSM-IV [12] as: intrusion, i.e. the persistent re-experience of the traumatic event with intrusive thoughts and images; avoidance, i.e. the persistent avoidance of stimuli associated with the traumatic event and “numbing” of general responsiveness; and hyperarousal, i.e. persistent symptoms of increased arousal) are particularly associated with poorer SQOL. The results were inconsistent. D’Ardenne and colleagues [8] reported that lower levels of avoidance were cross-sectionally associated with poorer quality of life. In a longitudinal study, Loncar and colleagues [11] found that changes in avoidance and hyperarousal clusters predicted changes in quality of life.

Our study assessed a large sample of people who met criteria for PTSD diagnosis following exposure to traumatic events during the war in former Yugoslavia. The data was collected within the CONNECT study, a multi-centre observational study on mental health consequences of war and migration. It assessed the prevalence of mental disorders in war-exposed people and the course of PTSD over time [13]. The prevalence rate of PTSD in Balkan residents was 20% [14] and in refugees 33% [15]. In Balkan residents, risk factors for being diagnosed with PTSD were older age, female sex, more traumatic experiences during and after the war, and unemployment [14]. In refugees, older age, a lower level of education, more traumatic experiences during and after the war, more migration-related stress, not feeling accepted by the host population, and having a temporary residence status were associated with having PTSD [15]. People with PTSD generated significantly higher health and social care costs [16]–[17].

In this longitudinal study we explored, in a sample size providing a high statistical power, whether and how changes in the levels of PTSD symptom clusters of intrusion, avoidance and hyperarousal are associated with changes in SQOL. We also assessed the direction of possible associations, i.e. whether symptom improvement leads to better SQOL or if improved SQOL results in symptom reduction. Associations between PTSD symptoms and SQOL were separately investigated in two samples: a representative sample of people who still lived in the post-conflict areas in five Balkan countries and a non-representative sample of refugees in three Western European countries. The direction of associations between PTSD symptom clusters and SQOL was explored by pooling data from the two groups in a common dataset and conducting a cross-lagged panel analysis of the reciprocal associations between PTSD symptom clusters and SQOL.

## Materials and Methods

The data was collected within the CONNECT study, a multi-centre observational study on mental health consequences of war and migration. The observational study (funded by the Research Directorate of the European Community) was conducted in war-affected communities in five Balkan countries (Bosnia and Herzegovina, Croatia, Macedonia, Kosovo, Serbia) and among refugees in three Western European countries (Germany, Italy, United Kingdom, i.e. the three countries in Europe with the highest numbers of immigrants in the 1990s).

CONNECT assessed the prevalence of mental disorders in war-exposed people and the course of PTSD over time [13]. A detailed description of the rationale and methods of the CONNECT project is available in previous publications [13]–[18].

### Ethics Statement

Written informed consent was obtained from all participants prior to the interview. The study was approved by the Royal Free Medical School Research Ethics Committee (REC reference number 04/QO501/118) and conducted in compliance with the Code of the Ethics of the World Medical Association as reported in the Declaration of Helsinki (2004) [19].

### Sampling Techniques and Participants

The assessments were carried out between January 2005 and November 2006. The global sample of the study included Balkan residents and refugees in Germany, Italy and United Kingdom.

A representative sample of residents in war-affected communities in five Balkan countries was recruited. Participants were chosen using a multi-stage probabilistic sampling frame and random walk approach in administrative regions that had been directly exposed to war activities. Areas and streets were randomly selected, and interviews were conducted in every fourth household. The eligible adult members of the identified households whose birthday was closest to the date of interviewing were asked to participate.

A sample of refugees from former Yugoslavia resettled in Germany, Italy and United Kingdom was also recruited in the study. For this sample a combination of random and non-random sampling approaches was adopted. In Germany and Italy, potential interviewees were identified through resident registers and snowball sampling. Due to the absence of such registers in the United Kingdom, potential interviewees were contacted through community organisations and snowball sampling.

The following inclusion criteria were applied: a) being born within the territory of former Yugoslavia; b) being between 18 and 65 years of age; c) having experienced at least one war-related potentially traumatic event and d) not having severe learning difficulty or mental impairment due to brain injury or other organic causes. Participants were excluded if they had experienced the last war-related event before 16 years of age.

Six hundred and sixty-five Balkan residents and 283 refugees met the criteria for PTSD on the MINI instrument at baseline. Out of these we attempted to follow up 620 Balkan residents (in Bosnia and Herzegovina the number of participants with baseline PTSD was too large to follow up all of them and 150 were randomly selected for re-interviews) and all of the refugees.

Numbers of eligible participants, those who were attempted to follow up, lost to follow up and interviewed in each country are reported in Table 1.

**Table 1:** Summary of recruitment and follow-up in each country.

Country	Eligible participants	Not in random sample	Attempted to follow-up	Lost to follow-up	Interviewed
Bosnia and Herzegovina	226	35	191	31	150^a^
Croatia	131	0	131	24	107
Kosovo	118	0	113	7	106
Macedonia	70	0	70	3	67
Serbia	120	0	120	20	100
Germany	140	0	135 b	21	114
Italy	56	0	56	16	40
UK	87	0	87	26	61
**Total**	**948**	**35**	**903** b	**148**	**745**

a 10 participants were excluded from the analysis due to duplicate ID numbers;

b 5 Albanian-speaking participants were excluded because no bi-lingual researcher was available.

### Procedures and Measures

Socio-demographic characteristics of participants including their age, sex, marital status, educational level, and current employment status were obtained using a brief structured questionnaire.

Mental disorders were assessed on the Mini International Neuropsychiatric Interview (MINI), a structured and validated diagnostic interview [20]. The symptom criteria in this instrument are assessed corresponding to the diagnosis Axis 1 of the DSM–IV [12]. The instrument has been used previously in war-affected and refugee groups [21]–[22].

The level of post-traumatic stress symptoms was measured on the Impact of Events Scale-Revised (IES-R) [23]. This self-report instrument assesses 22 intrusion, avoidance and hyperarousal symptoms within the last 7 days with regard to a specific traumatic event. Each IES-R item is rated on a five-point scale of distress (0–4).

Manchester Short Assessment of Quality of Life (MANSA) was used to assess subjective quality of life [24]. The MANSA contains 12 items on satisfaction with life in general and with various life domains (employment, financial situation, friendships, leisure activities, accommodation, personal safety, living situation, sex life, relationships with family, physical health, mental health) which are rated on a scale from 1, could not be worse, to 7, could not be better. The MANSA has shown good psychometric properties: Cronbach’s alpha for MANSA items scores was 0.74 and MANSA mean score had a strong correlation with another established instrument for SQOL measurement, the Lancashire Quality of Life Profile, which has a much higher number of items [24]. The mean score of the MANSA was taken as a measure of SQOL.

Post-traumatic symptoms and SQOL were reassessed after 12 months.

At both time points, interviews were conducted face-to-face by 33 trained interviewers, who were qualified psychologists, psychiatrists, sociologists or anthropologists. All those instruments for which there had been no validated translations in the relevant languages were translated and back-translated into English.

### Statistical Analysis

Descriptive statistics were used to summarise the characteristics of participants who completed follow-up in the two samples. The differences in socio-demographic and clinical characteristics of participants who were followed-up and those who dropped out from the study were assessed using two-tailed X^2^ tests and analysis of variance (ANOVA) tests, depending on the type of data. For one hundred and thirty-five participants who completed the follow-up (18.1%) a maximum number of two items was missing from IES-R and/or MANSA. To avoid potential problems of a list-wise deletion of incomplete cases [25] a multiple imputation procedure was conducted [26].

Two-tailed paired t-tests were used to compare MANSA total score and IES-R subscales scores between baseline and follow-up.

Univariable linear regression models were used to explore, in the two groups (Balkan residents and refugees), associations between follow-up scores of PTSD symptom clusters of intrusion, avoidance and hyperarousal (IES-R) and SQOL. The univariable association of each cluster with SQOL at follow-up was adjusted for the scores of the given symptom cluster and SQOL at baseline. This type of analysis is commonly used for exploring changes over time although strictly speaking it does not use change scores [5].

Multivariable models were tested to adjust associations between symptom clusters and SQOL for confounding factors. With SQOL at follow up being the dependent variable again, scores of the three symptom clusters at follow-up were independent variables. The associations were adjusted for baseline scores of the three symptom clusters and SQOL as well as socio-demographic and clinical variables that were significantly associated with SQOL at follow up. As potentially relevant sociodemographic and clinical variables, age, gender, years in education, marital status, unemployment, living alone, comorbidity with other mental disorders, number of years since the exposure to traumatic events and country of residence were tested.

A two-wave cross lagged panel analysis was used to assess the direction (temporal ordering) of the association of the symptom clusters, which significantly covaried with SQOL over time, and SQOL. This method has been widely used to establish the direction of relationships between psychiatric symptoms and environmental factors [27]–[28]. The cross-lagged panel analysis was carried out on the pooled dataset in order to achieve an adequate sample size. The Cronbach’s alpha values of all the variables used (MANSA total score at baseline and follow-up; IES-R hyperarousal subscale at baseline and follow-up) were calculated to ensure that their internal consistency was sufficiently high to be included in the model, without creating latent variables.

Since we found a correlation between hyperarousal symptoms and SQOL (p<.01) at baseline and at follow-up in Pearson’s tests, the variables measured at the same time point were allowed to covary, resulting in a fully saturated model [28].

Stata 12 for Windows was used for all data analyses [29].

## Results

A summary of recruitment and follow up is reported in Table 1.

Seven hundred and forty-five subjects diagnosed with PTSD were included in the analysis, i.e. 530 Balkan residents (follow up rate: 85.5%) and 215 refugees (follow-up rate: 76%). The diagnosis was established according to the MINI. Rating agreement among interviewers was assessed for the MINI in 2 mock interviews. An agreement on an item was reached when all interviewers gave it the same answer. Among 251 items, the mean agreement rate across 2 sessions was 90.2%.

Overall, re-interviewed participants were significantly more often female (56% vs. 41%, Χ^2^ = 11.475, df = 1, p<.001), had experienced fewer traumatic war events (6.5 SD = 3.4 vs. 7.6 SD = 3.8, F = 14.210, df = 1.902, p<.001), had less often participated in war activities (22% vs. 39%, Χ^2^ = 12.253, df = 1, p<.001), and had experienced the most traumatic war event a shorter time before the study (9.1 SD = 3.2 vs. 10.0 SD = 3.1, F = 17.854, df = 902, p<.001). No significant differences in baseline PTSD symptoms and SQOL levels were found.

The main socio-demographic and clinical characteristics of the total sample and of the Balkan residents’ and refugees’ groups are summarized in Table 2.

**Table 2:** Patients’ characteristics.

	Total sample	Balkan residents	Refugees
	(n = 745)	(n = 530)	(n = 215)
Age, mean (sd)	45.4 (10.8)	45.6 (11.1)	44.8 (10.2)
Gender, female, n(%)	420 (56.4)	296 (55.8)	124 (57.7)
Education in years, mean (sd)	10.4 (3.7)	10.2 (3.6)	10.8 (4.0)
Married/partnership, n(%)	529 (71.0)	364 (68.7)	165 (76.7)
Living alone, n(%)	70 (9.4)	48 (9.1)	22 (10.2)
Unemployed, n(%)	417 (56.0)	273 (51.5)	144 (67.0)
**MANSA total score**			
Baseline, mean (sd)	4.1 (1.0)	4.1 (1.0)	4.2 (1.0)
Follow-up, mean (sd)	4.3 (0.9)	4.2 (1.0)	4.4 (0.8)
**IES-R intrusion subscale**			
Baseline, mean (sd)	2.6 (0.9)	2.5 (0.9)	2.8 (0.9)
Follow-up, mean (sd)	2.1 (1.1)	2.0 (1.0)	2.3 (1.2)
**IES-R hyperarousal subscale**			
Baseline, mean (sd)	2.5 (1.0)	2.5 (1.0)	2.7 (1.0)
Follow-up, mean (sd)	2.0 (1.1)	2.0 (1.1)	2.2 (1.3)
**IES-R avoidance subscale**			
Baseline, mean (sd)	2.3 (0.9)	2.2 (0.8)	2.4 (0.9)
Follow-up, mean (sd)	1.9 (1.0)	1.8 (1.0)	2.0 (1.0)

At the one year follow-up, the levels of SQOL were significantly improved in both samples and the scores of the IES-R subscales were significantly reduced (p<.001 for all paired t-tests).

Linear regression models for association of changes in PTSD symptom clusters and SQOL in Balkan residents and refugees are reported in Table 3 and Table 4, respectively.

**Table 3:** Univariable^a^ and multivariable linear regression modelsb,c,d describing the relationship between subjective quality of life and PTSD symptoms in residents in war-affected countries (n = 530).

Univariable models				Multivariable model		
	B	B (95% CI)	p	B	B (95% CI)	p
IES-R subscales						
Intrusion	−.361	−.445 to −.277	<.001	−.045	−.168 to.079	.478
Hyperarousal	−.367	−.445 to −.288	<.001	−.221	−.334 to −.109	<.001
Avoidance	−.291	−.308 to −.201	<.001	.028	−.062 to.119	.540

a Controlled for MANSA score at baseline and specific IES-R subscale at baseline.

b Dependent variable: MANSA score at follow-up.

c Independent variables: IES-R subscales (intrusion, hyperarousal, avoidance) at follow-up.

d Variables controlled for in the multivariable model: MANSA and IES-R subscales score at baseline, gender, years elapsed since the end of the conflict.

**Table 4:** Univariable^a^ and multivariableb,c,d linear regression models describing the relationship between subjective quality of life and PTSD symptoms in refugees in western countries (n = 215).

Univariable models				Multivariable model		
	B	B (95% CI)	p	B	B (95% CI)	p
IES-R subscales						
Intrusion	−.184	−.285 to −.082	<.001	.071	−.096 to.238	.403
Hyperarousal	−.239	−.334 to −.143	<.001	−.242	−.397 to −.087	.002
Avoidance	−.264	−.354 to −.134	<.001	−.033	−.187 to.121	.675

a Controlled for MANSA score at baseline and specific IES-R subscale at baseline.

b Dependent variable: MANSA score at follow-up.

c Independent variables: IES-R subscales (intrusion, hyperarousal, avoidance) at follow-up.

d Variables controlled for in the multivariable model: MANSA and IES-R subscales score at baseline, gender, years elapsed since the end of the conflict.

In the univariable models, reduction in all symptom clusters levels was associated with improvements in SQOL. Besides symptoms, only gender and number of years since the end of the exposure to traumatic events had a significant association with SQOL at follow up. These variables were entered in the multivariable model, adjusted for baseline scores of all symptom clusters and SQOL. In the multivariable models, only changes in hyperarousal symptoms were correlated with SQOL changes. The results were consistent in both samples.

The values of tests for multicollinearity for these multivariable models were in the acceptable range (all values of tolerance were above 0.1 and all values of VIF were less than 5).

The four variables used in the cross-lagged panel analysis (hyperarousal symptoms and SQOL both at baseline and at follow up) had a good internal consistency. Cronbach’s alpha values were 0.861 for IES-R hyperarousal subscale at baseline, 0.910 for IES-R hyperarousal subscale at follow-up, 0.810 for SQOL at baseline and 0.857 for SQOL at follow-up. These variables were, therefore, used in the model as measured variables without a need for creating latent variables.

**Figure 1:**
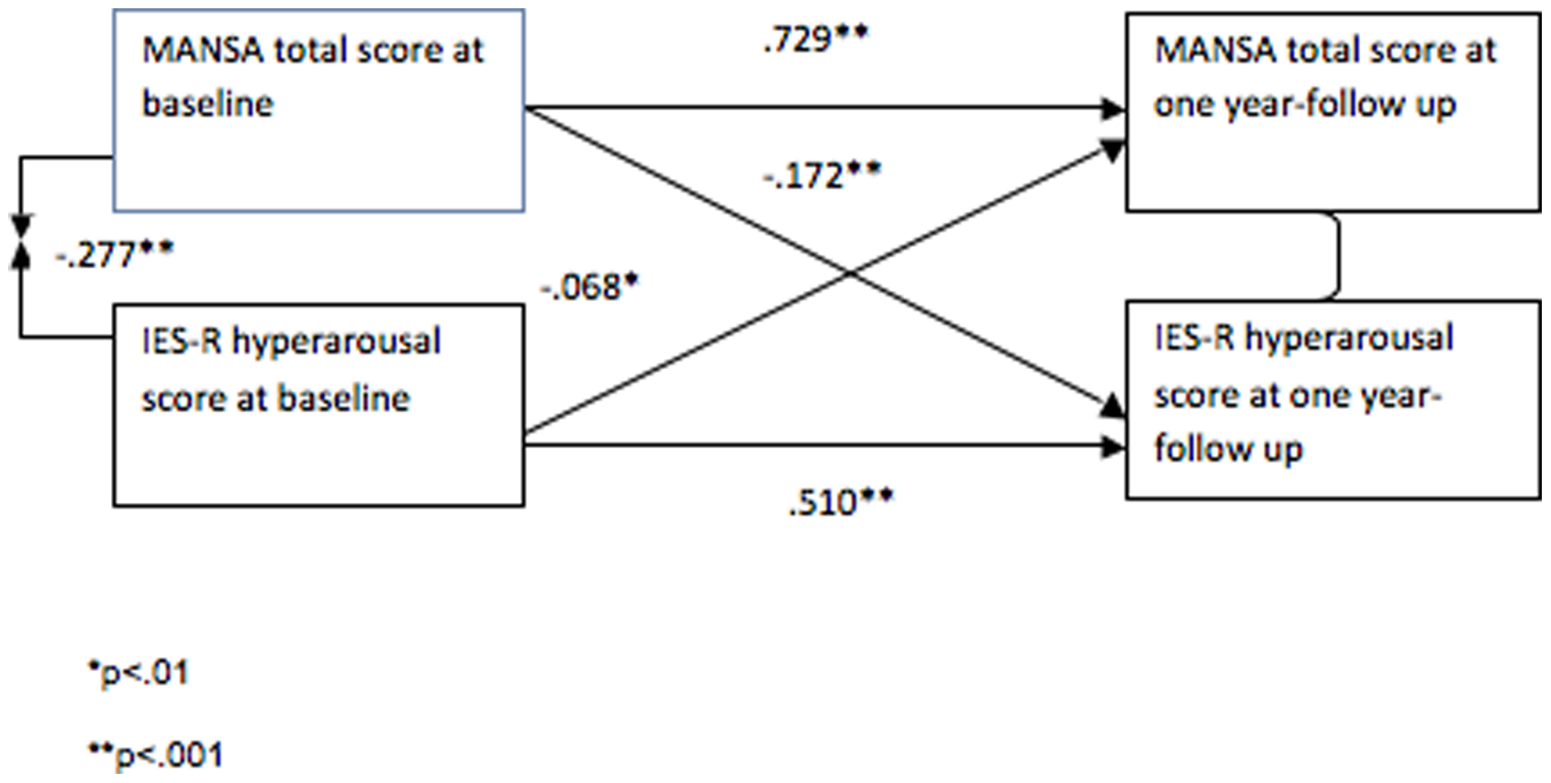
Cross-lagged panel analysis of relationship between hyperarousal and subjective quality of life in PTSD (n = 745).

Figure 1 shows the results of the two-wave cross lagged panel analysis.

SQOL and IES-R hyperarousal subscales scores had a significant inverse correlation at baseline (Pearson test’s value: −.286, p<.01) and at follow-up (Pearson test’s value: −.430, p<.01), hence the variables measured at the same time point were allowed to covariate in the model.

The association between hyperarousal symptoms and SQOL was bidirectional. A statistically significant negative beta coefficient was found for the path from hyperarousal symptoms at baseline to SQOL at one year-follow up (b = −.068, p<.01). Also the path for the reverse temporal ordering, from SQOL at baseline to hyperarousal symptoms at one year-follow up, was statistically significant (b = −.162, p<.001).

## Discussion

### Main Results

Changes in hyperarousal symptoms were associated with changes in SQOL over time in both univariable and multivariable models, controlled for other symptom clusters and main socio-demographic and trauma-related characteristics. Changes in intrusion and avoidance symptoms are linked with SQOL changes in univariable models only, in which they may just reflect the global severity of the PTSD symptomatology.

A cross-lagged panel analysis suggested a reciprocal influence between hyperarousal and SQOL. A reduction of hyperarousal symptoms may lead to improved SQOL, and – vice versa – an improved SQOL may also result in reduced PTSD symptoms.

### Strenghts and Limitations

This is the largest longitudinal study to date assessing SQOL in people with PTSD. The sample of Balkan residents can be considered representative for war affected people in the participating countries. Consistent assessment methods were used across eight countries and the samples included both civilians and people who actively participated in the war. Standardized instruments for measuring PTSD symptoms and SQOL were administered face to face by trained researchers. Interrated reliability between research workers was excellent (90%). Findings in the two samples were consistent, although they differ in their characteristics and live in a different context.

There are also limitations: 1) Refugees’ sample cannot be considered representative. However, the data protection legislation and the absence of complete data registers in the Western European countries do not allow for fully representative sampling of refugees, and the results in refugees are consistent with those in Balkan residents, indicating an overall validity; 2) The associations identified in this analysis might be explained by confounding factors that have not been assessed in the study (i.e. genetic and biological factors); 3) The cross-lagged analysis shows how different scores follow each other, but does not establish causality; 4) Not all participants interviewed at baseline were followed up and there is no data on the changes of PTSD symptoms and SQOL in those who were not followed up. We cannot rule out that a selection bias may have influenced the results, since subjects who dropped out were more frequently male and with a more intense exposure to war events. However, the levels of PTSD symptoms and SQOL at baseline did not differ between drop-out and people re-interviewed at follow-up; 5) PTSD symptoms are known to fluctuate over time and this might have influenced the results [30].

### Comparison with Literature

In our study, high levels of hyperarousal symptoms were associated with lower SQOL in people with war-related PTSD. Hyperarousal was the only symptom cluster that showed an association with SQOL when controlling for all the symptom clusters in multivariable models. The association of higher levels of hyperarousal symptoms with poorer SQOL has already been reported in smaller samples of people with PTSD [9]–[11]. This association may be explained in light of the specific types of symptoms included in hyperarousal cluster. Sleeping difficulties and recurrent nightmares may significantly reduce levels of satisfaction with physical and psychological health and are particularly resistant to treatment [31]–[32]. Hypervigilance and irritability could pose difficulties in family and social relationship [33] and difficulties in concentration may reduce work and personal functioning [34]. The findings of this study show that the improvement of these very distressing symptoms is associated with better SQOL, independently from changes in other symptom clusters.

On the other hand, in our study, avoidance and intrusion levels did not show a significant association with SQOL in multivariable models. Avoidance may even work as a coping strategy, temporarily reducing discomfort and limiting severe dissatisfaction with quality of life [35]–[36]. Similarly, people with PTSD might deal with the recurrence of intrusive image or thoughts by avoiding “triggering” events or conditions, trying to distract themselves or even adopting unhealthy behaviours like alcohol and benzodiazepines abuse [37]; this may reduce the impact of intrusion symptom cluster on subjective quality of life. The enduring discomfort related to high levels of hyperarousal symptoms and the related generalized anxiety may be more difficult to cope with than the more specific anxiety captured in some intrusion and avoidance symptoms [38] and lead to a negative impact on SQOL.

Finally, our results suggest that a poorer SQOL, which may be due to psychosocial factors (unemployment, social isolation, economic problems, etc.), might influence the level of hyperarousal symptoms. The impact of poor living conditions on the level of anxiety symptoms has already been described in PTSD [39]–[40]. As documented in patients with personality disorders [41], the sense of safety has a strong influence on SQOL. Precarious living conditions may be at least partially responsible for the persistence of higher levels of hyperarousal symptoms. On the other hand, a feeling of being unsafe, as reflected in hyperarousal symptoms, might impair a positive perception of living conditions and, therefore, reduce SQOL scores. SQOL and hyperarousal symptoms may reflect different but related aspects of feeling unsafe and threatened.

### Implications

Taking into account the association between hyperarousal symptoms and SQOL, hyperarousal symptoms should be a primary target for treatment aimed at improving SQOL in war related PTSD. Some evidence suggests that selective serotonin reuptake inhibitors, mood stabilizers and atypical anti-psychotics may be effective in reducing hyperarousal symptoms [42]. Sympatholytic drugs appear to be particularly useful as an add-on therapy for treatment-resistant hyperarousal symptoms such as nightmares [32]. Furthermore, several studies have documented the positive effects of psychological therapies such as trauma-focused cognitive behavioral therapy, eye movement desensitization and reprocessing [43], and in particular, of relaxation training on hyperarousal [44].

Our findings indicate a bidirectional association between hyperarousal symptoms and SQOL. Whilst symptom reduction may improve SQOL, improvements of SQOL may result in reduced hyperarousal symptoms. One can speculate as to whether social interventions improving life conditions of people with PTSD might ameliorate their hyperarousal symptoms. In fact, social support has been associated with an higher likelihood of recovery in PTSD patients [42], [45] whereas the presence of specific stressors, such as those related to migration, is associated with higher PTSD symptom levels [15]. Identifying and meeting the psychosocial needs of people with PTSD may be important for improving SQOL and, as a consequence, lead to a remission of hyperarousal which reflects “core” symptoms of PTSD.

### Conclusions

The subjective quality of life of individuals with war related PTSD is particularly associated with their levels of hyperarousal symptoms. Experimental studies are required to explore whether the associations found in this large observational study reflect causal relationships that translate into direct treatment recommendations. These studies should test whether treatments targeting hyperarousal symptoms have a beneficial effect on SQOL, and whether effective social interventions specifically reduce hyperarousal symptoms.
